# Comparisons of a Multi-Frequency Bioelectrical Impedance Analysis to the Dual-Energy X-Ray Absorptiometry Scan in Healthy Young Adults Depending on their Physical Activity Level

**DOI:** 10.1515/hukin-2015-0063

**Published:** 2015-10-14

**Authors:** Julien Verney, Chloé Schwartz, Saliha Amiche, Bruno Pereira, David Thivel

**Affiliations:** 1 Clermont University, Blaise Pascal University, EA 3533, Laboratory of the Metabolic Adaptations to Exercise under Physiological and Pathological Conditions (AME2P), Clermont-Ferrand, France.; 2 CRNH-Auvergne, Clermont-Ferrand, France.; 3 Clermont-Ferrand University hospital, Biostatistics unit (DRCI), Clermont-Ferrand, France.

**Keywords:** Fat Mass, Fat-Free Mass, Bio-impedance, DXA, physical activity level

## Abstract

This study aimed at comparing BIA and DXA results in assessing body composition in young adults depending on their physical activity level. Eighty healthy 19–30 years old subjects were enrolled and their body composition (Fat Mass and Fat-Free Mass) was assessed by dual-energy X-ray absorptiometry (DXA) and by a newly developed Bioelectrical Impedance Analyzer (BIA - Tanita MC780). A seven-day physical activity level was assessed using a 3-axial accelerometer. DXA-FM% and BIA-FM% were correlated (p<0.001; r= 0.852; ICC [IC95%]: 0.84 [0.75 – 0.90]; concordance coefficient: 0.844). DXA-FFM and BIA FFM were correlated (p<0.001; r=0.976; ICC [IC95%]: 0.95 [0.93 – 0.97], concordance coefficient: 0.955). DXA and BIA measurements of FM% and FFM were highly correlated in both boys and girls regardless of the physical activity level. Compared with DXA scans, newly developed bioelectrical impedance analyzers provide satisfactory fat mass and lean mass measures in healthy young women and men, despite their physical activity level.

## Introduction

Recent years have seen an important and rapid development of methods and technologies dedicated to the assessment of body composition and especially to the quantification of total and regional body fat (Ayvaz, 2011). This improvement is mainly due to a growing interest in body fat estimation and relates to the alarming progression of overweight, obesity, and their metabolic complications. A large range of methods are nowadays available to assess or directly measure body composition, from field testing to laboratory or clinical technologies. Estimations derived from anthropometric measurements, such as skinfolds or a Body Adiposity Index have proved their validity (Begman et al., 2011; Bergman, 2012; Thivel et al., 2014), but remain indirect estimations. At the opposite, Magnetic Resonance Imagery (MRI) or Dual-energy X-ray absorptiometry (DXA) offer high accuracy and precision, but remain expensive and of limited access (clinical or laboratory settings). Since body composition assessment is of particular interest in both clinical and training fields, it seems necessary to use accessible tools able to provide accurate and validated results, almost as precise as gold standard methods (such as DXA or MRI). Bioelectrical impedance analyses (BIA) have been developed to offer a reliable alternative between direct and indirect measurements. Although this technology achieves great success in various domains (dietitians, clinical setting, training centers, etc.), scientific results remain contradictory concerning its ability to properly measure body fat compared with reference methods (Kitano et al., 2001; Esco et al., 2014). While some studies report that BIA offers accurate estimates of body fat percentage (Lloret et al., 2011; Jebb et al., 2007), some others observed overestimated percentage of body fat (Ravaglia et al., 1999), or underestimated values (Erselcan et al., 2000; Wattanapenpaiboon, 1998). Beyond the simple estimation of body fat, some authors have shown different limits of agreement when comparing body composition (fat mass and fat-free mass) between BIA and DXA (Spencer et al., 2003; Demura et al., 2005; Esco et al., 2014). Assessing body composition using BIA remains highly difficult because of the strict conditions required to perform the test. It is indeed very difficult in practice to perform analysis in fasted patients, under strictly controlled ambient temperature and hydration status. To overpass such methodological and practical limitations, recent devices based on the BIA technology have been developed that allow measurements at any time of the day, without nutritional or physical activity constraints.

The first aim of this work was to compare the use of a newly developed bioelectrical impedance analyzer (BIA) with a DXA scan when assessing body composition in healthy young adults. We also questioned whether or not the physical activity level of individuals could affect the accuracy of their body composition assessment using BIA.

## Material and Methods

### Population and design

A total of 80 healthy young adults aged 19 to 30 years old were recruited among students and academics from the local university. All of them had a healthy Body Mass Index (BMI) between 20 and 25 kg/m^2^ (according to the World Health Organization). All the participants were informed of the purpose of the study, received information sheets and signed consent forms. Once included in the study, the participants visited the laboratory for assessment of their body composition and anthropometric measurements. They were also asked to wear a tri-axial accelerometer for 7 days to objectively assess their physical activity level. Of the initially 80 enrolled participants, complete data for body composition (DXA and BIA) were obtained from 71 subjects (36 males and 35 females). Fifty-five accepted to wear the accelerometer for 7 days and clean data for physical activity level were obtained from 41 subjects.

### Anthropometric measurements and body composition assessment

A digital scale was used to measure body mass to the nearest 0.1 kg, and barefoot standing height was assessed to the nearest 0.1 cm by using a wall-mounted stadiometer. The Body Mass Index (BMI) was calculated as body mass (kg) divided by height squared (m^2^). Body composition was then assessed on the same occasion by dual-energy X-ray absorptiometry and impedance analysis as follows:

### Dual-energy X-ray absorptiometry

Percentage Fat Mass (FM) and absolute Fat mass and Fat Free Mass (FFM) were assessed using dual-energy X-ray absorptiometry (QDR4500A scanner, Hologic, Waltham, MA, USA). From DXA analysis, abdominal fat mass (visceral and subcutaneous tissues) was determined manually by an experienced technician by drawing a rectangular box around the region of interest between the vertebral bodies of L1 and L4. The upper limit was set with a horizontal line going through the T12/L1 disk space and the lowest limit was set with a horizontal line going through the L4 and L5 disk space (Glickman et al., 2004; Aucouturier et al., 2009). Data were analyzed with Hologic QDR software for Windows (version 12.6). This method had been previously used and validated (Glickman et al., 2004) among different populations (Thivel et al., 2014; Aucouturier et al., 2009).

### Bioelectrical impedance

The bioelectrical impedance measurements were performed with the Tanita MC780 multi frequency segmental body composition analyzer. This consists in a stand-alone unit where the subject has to step on barefoot (standard mode). Information concerning the subject (age, gender and height) are entered by the experimenter. Once body mass has been assessed by the scale, the subject has to take grips in both hands (alongside his body) during the impedance measure (Hand to foot BIA). A full segmental analysis is performed in less than 20 s. Segmental fat mass and fat-free mass values are indicated by the end of the analysis on the digital screen (trunk, left and right arms and legs); as well as total body fat, fat-free mass and water. A visceral index (from 1 to 55) is also proposed as an estimation of visceral fat. Total fat and Fat-Free Mass, as well as the visceral adiposity index were reported by the investigator into an excel sheet for statistical treatment.

### Physical activity level

The participants were asked to wear a portable Actigraph during 7 consecutive days to assess their physical activity level. The Actigraph GT3X activity sensor consists of a three-axial acceleration sensor (adxl335, Analog Devices, Boston, USA; range: ± 3 g; sampling rate: 30 Hz; resolution: 12 bit). The actigraph weighs 27 grams and measures 3.8 × 3.7 × 1.8 cm. This device can be worn either at the wrist or hip and can record activity for up to 21 consecutive days. In the present work, the participants were asked to wear the accelerometer at their hip for 7 days. They were instructed to remove the accelerometer when water contacts were possible (shower or swimming pools). If they removed the device to swim, they had to indicate the exact day and hour and to describe their session. The recorded data were saved as activity counts on a 4 MB flash memory and transferred to the computer via a standard USB 2.0 interface. Using the information from the Actigraphs, a 24 h physical activity level was estimated for each participant. The impact of PAL was analyzed following two different approaches: as an adjustment factor and by dividing the sample by 2 according to the PAL obtained (50% above and 50% under the median PAL).

### Statistical analysis

Statistical analysis was performed using Stata 13 software (StataCorp LP, College Station, TX, US). The tests were two-sided, with a type I error set at α = 0.05. To study the agreement between two measurement methods (BIOA TANITA MC780 and DXA) for MGtot and FFMkgtot, statistical analyses considered correlation coefficients (Pearson or Spearman according to statistical distribution), the concordance Lin coefficient, the intra-class correlation coefficient (ICC estimated using random effects model) and the Bland and Altman representation. For Visceral rating, only the correlation analysis was applied because the scale was not identical for the two measurement methods. Before considering subgroup analyses according to PAL, multiple linear regressions were proposed taking into account PAL as an adjustment factor. The regression coefficients, associated 95% confidence intervals and r^2^ were studied. Then, with regard to the statistical distribution of PAL, previous analyses were reproduced for PAL ≤ ≥ median. In addition, a sensitivity analysis was performed to complete these analyses and to ensure that the results were the same by varying the PAL discriminating threshold (data not shown).

## Results

The mean age of the whole sample (n=71) was 22.7 ± 3.5 years old with mean body mass of 65.8 ± 11.6 kg and a Body Mass Index of 21.61 ± 4.81 kg/m^2^. The PAL of the participants ranged from 231585 to 1014887 counts/min/24h. Table 1 details the body composition values obtained using both DXA and BIA for both genders and the whole sample. The percentage of Fat Mass and the absolute FFM (kg) obtained were not statistically different between DXA and BIA in females, males and for the whole group.

**Figure 1:**
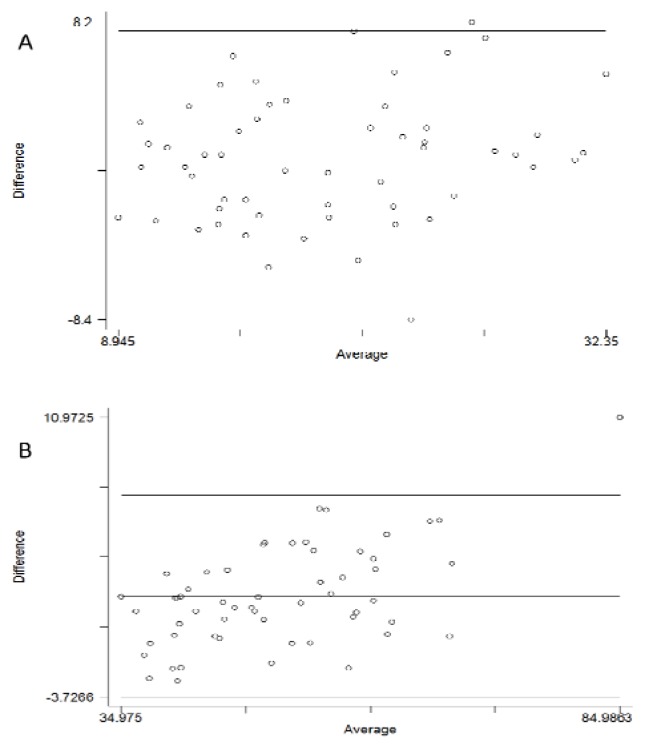
Bland-Altman pairwise comparison between the percentages of total body fat (%FM) assessed by DXA and BIA (A); and between Fat-Free Mass assessed by DXA and BIA (B). (DXA: Dual X-ray absorptiometry; BIA: Bioelectrical Impedance Analysis)

There was a significant correlation between the percentage of fat mass measured by DXA and the one measured by BIA (p<0.001; r= 0.852), with an intra-class correlation coefficient *ICC* [IC_95%_] of 0.84 [0.75 – 0.90] and a concordance coefficient of 0.844. The correlation coefficient considering the physical activity level between BIA-FM% and DXA-FM% was 0.84. The Figure 1A shows the Bland and Altman analysis for DXA-FM% and BIA-FM%. There was a significant correlation between total fat-free mass in kilograms assessed by DXA (DXA-FFM) and BIA (BIA-FFM) (p<0.001; r=0.976). The intra-class correlation coefficient *ICC* [IC_95%_] was 0.95 [0.93 – 0.97] with a concordance coefficient of 0.955. The correlation coefficient considering the physical activity level between BIA-FFM and DXA-FFM was 0.95. The plots of the Bland-Altman analysis are shown in Figure 1B. Although correlation analysis between central FM percentage assessed by DXA and the visceral index proposed by the BIA reached the level of significance (p<0.05), the correlation coefficient remained very low (r=0.275; with PAL as an adjustment factor: r=0.240). The same results were obtained when considering males and females separately.

Considering only the 41 participants that wore the accelerometers for 7 days, DXA-FM% and BIA-FM% were significantly correlated (p<0.001; r = 0.839) with a concordance coefficient of 0.84 and the ICC [IC_95%_] of 0.84 [0.73–0.91]. DXA-FFM and BIA-FFM were also significantly correlated (p<0.001; r = 0.935), with a concordance coefficient of 0.96 and the ICC [IC_95%_] of 0.96 [0.92–0.98]. The correlation coefficient between the percentage of central fat mass assessed by DXA and the visceral index proposed by BIA was r= 0.36 (p<0.05).

When considering 50% of the sample that presented a PAL under the median, DXA-FM% and BIA-FM% were significantly correlated (p<0.001; r = 0.835) with a concordance coefficient of 0.84 and the ICC [IC_95%_] of 0.84 [0.66–0.93]. DXA-FFM and BIA-FFM were also significantly correlated (p<0.001; r = 0.938), with a concordance coefficient of 0.93 and the ICC [IC_95%_] of 0.96 [0.84–0.97]. The correlation coefficient between the percentage of central fat mass assessed by DXA and the visceral index proposed by BIA was r= 0.28 (p<0.05).

In 50% of the sample with a PAL above the median value, DXA-FM% and BIA-FM% were significantly correlated (p<0.001; r = 0.833), with a concordance coefficient of 0.84 and the ICC [IC_95%_] of 0.84 [0.67–0.93]. DXA-FFM and BIA-FFM were also significantly correlated (p<0.001; r = 0.972), with a concordance coefficient of 0.97 and the ICC [IC_95%_] of 0.96 [0.93–0.99]. The correlation coefficient between the percentage of central fat mass assessed by DXA and the visceral index proposed by BIA was r= 0.38 (p<0.05). The same results were obtained when considering males and females separately.

## Discussion

The aim of the present work was to compare total body fat, visceral fat and lean body mass assessed by Dual energy X-ray absorptiometry and a newly developed bio-electrical impedance (Tanita MC780) among healthy normal weight young adults, depending on their objectively measured level of physical activity.

Our results underline a high level of correlation between the two methods (DXA and BIA) when measuring total body fat percentage (r=0.852, ICC: 0.84) with a high level of concordance (0.844). Previous studies have compared the use of BIA with DXA or MRI as gold standards to measure body fat percentage in various populations, and most of them found that the bio-electrical impedance was a less accurate method (Esco et al., 2014; Velazquez-Alva et al., 2014; Wang et al., 2013; Völgyi et al., 2008), providing underestimated (Erseclan et al., 2000; Wattanapenpaiboon et al., 2000) or overestimated (Ravaglia et al., 1999) values. Importantly, it has to be kept in mind than electrical impedance in evaluation of body composition is highly dependent on the hydration state of the body as the resistance of body tissues especially muscle changes according to the mineral and water content. The fact that our sample was mainly composed of healthy normal-weight young adults (19–30 years old) certainly explains why we found an important agreement between the two methods. Indeed, our whole sample presents a low and relatively homogenous percentage of total body fat (19.2 ± 6.5 % according to the DXA scan). Velasquez-Alva et al. (2014) recently showed in young adults that the bias of measure between the two methods raises with increased body fat percentage. In other words, the higher is the percentage of body fat, the higher is the discrepancy between DXA and BIA. Our results also show a high level of agreement between BIA and DXA methods for the assessment of Fat Free Mass. This result is in line with the recent paper of Wang et al. (2013) concluding that BIA is an accurate method to measure skeletal muscle mass and fat free mass in middle aged (mean age of 48 years old) men and women (Wang et al., 2013). However, Velasquez-Alva et al. (2014) and Esco et al. (2014) found slightly nuanced results with wide limits of agreement between the two methods despite close means in normal weight and athletic women. As previous BIA devices, the newly developed TANITA MC780 proposes a visceral adiposity index (ranking from 1 to 55, 1 being the lowest level of visceral adiposity). Although the DXA scan does not provide a direct measure of visceral fat, it allows to estimate central adiposity (Glickman et al., 2004; Aucouturier et al., 2009). The present analysis revealed that the BIA-proposed visceral adiposity index was not accurate when compared with the DXA-estimated central adiposity percentage, which is in accordance with previously published results (Wang et al., 2013). Pietilainen et al. (2013) compared the use of a single frequency BIA with DXA and MRI and found that although BIA overestimated visceral fat compared with MRI in obese adults (especially in males), it remained a good cross-sectional measure of visceral fat, however, was not accurate in investigating longitudinal changes. The validity of the BIA-derived index has been shown to be highly dependent on the subject’s weight status, as it underestimates visceral fat in people with a BMI below 35 kg/m^2^ and overestimates it when their BMI overpasses 35 kg/m^2^ (Berker et al., 2010).

Although the importance of weight status when using BIA has been widely studied and highlighted, less data are available regarding the impact of the individuals’ physical activity level (while BIA is often used among athletes). As most of the BIA devices propose to perform the analysis using a “standard” or “athlete” model, Swartz et al. (2002) compared the use of both modes among active, moderately active and low active 18 to 35 years old healthy men. Using a foot-to-foot BIA, the authors found that the body fat percentage was not different when assessed following the standard or athlete mode in people with a low activity level, while it was higher using the athlete mode in both moderately active and active subjects (Swartz et al., 2002). Similarly, they found that Fat-Free Mass was higher using the athlete mode regardless of the subjects’ physical activity level (Swartz et al., 2002). Although their work was the first to question the importance of the PAL when assessing body composition by BIA, they did not compare the obtained values with a gold standard measure and they used a highly subjective method to assess PAL (questionnaires). With the same methodological limits (questionnaires to assess PAL), Volgyi et al. (2008) compared the results from BIA and DXA and found that the fat mass percentage was underestimated using BIA in people with a low physical activity level (Volgyi et al., 2003). In their recent work, Gaba et al. (2014) were the first to compare the use of BIA and DXA depending on objectively measured PAL. The results showed a high level of correspondence between the two methods when assessing FM% and FFM in postmenopausal women with different physical activity levels (Gaba et al., 2014). Although they used an objective method to assess PAL, the selected accelerometers were uni-axial and may have underestimated PAL. In the present work, the participants were asked to wear a tri-axial Actigraph accelerometer for 7 days. According to our results, whatever the individual’s level of physical activity, the Tanita MC780 provides an accurate measure of both FM% and FFM, without gender differences. Although BIA devices propose an “athlete” mode, it remains difficult for the experimenter or the subject to estimate whether or not this mode should be applied (except for highly active individuals). Our data suggest then that in healthy young adults presenting a wide range of the physical activity level, the standard mode can be used regardless of the level of physical activity to accurately assess body composition.

The relatively large sample size (n=71) represents one of the methodological strengths of the present comparison between the two devices, however, the reduced number of participants that properly wore the accelerometers (n=41) might be a limitation. Once more, the relatively homogenous nature of our sample may explain the fairly good correspondence between BIA and DXA, which suggests the need for complementary studies involving people from other weight status. The main strength of the present work remains the use of a highly objective method to assess the participants’ physical activity using a 3-dimensional accelerometer.

The newly developed BIA Tanita device (MC780) provides then an accurate tool to measure total body fat percentage and Fat Free Mass, but not visceral fat, in healthy young males and females, regardless of their level of habitual physical activity. This method can be recommended to the physical training staff or personal coaches as part of their training programs, however, further investigations are needed to test its validity among particular populations such as overweight or obese subjects.

## Figures and Tables

**Table 1:** Body composition results obtained using both DXA and BIA for both genders and the whole sample

	Females		Males		Whole group	
	*DXA*	*BIA*	*DXA*	*BIA*	*DXA*	*BIA*
FM %	23.7 ± 5.5	22.1 ± 5.1	14.3 ± 3.2	14.8 ± 4.4	19.2 ± 6.5	18.4 ± 6.0
Central FM %	21.8 ± 6.4	/	15.9 ± 4.2	/	18.9 ± 6.2	/
FFM kg	44.1 ± 5.22	43.2 ± 4.2	60.9 ± 8.2	58.0 ± 7.3	52.3 ± 10.7	50.2 ± 9.5
Visceral Index	/	1.3 ± ,0.7	/	2.3 ± 2.1	/	1.81 ± 1.6

%: percentage; DXA: Dual-energy X-ray absorptiometry; BIA: Bioelectrical Impedance Analysis; kg: kilograms.

## References

[b1-jhk-47-73] Aucouturier J, Meyer M, Thivel D, Taillardat M, Duche P (2009). Effect of android to gynoid fat ratio on insulin resistance in obese youth. Arch Pediatr Adolesc Med.

[b2-jhk-47-73] Ayvaz G (2011). Methods for body composition analysis in adults. Open Obesity.

[b3-jhk-47-73] Bergman RN (2012). A better index of body adiposity. Obesity (SilverSpring).

[b4-jhk-47-73] Bergman RN, Stefanovski D, Buchanan TA, Sumner AE, Reynolds JC, Sebring NG, Xiang AH, Watanabe RM (2011). A better index of body adiposity. Obesity (SilverSpring).

[b5-jhk-47-73] Berker D, Koparal S, Işik S, Paşaoğlu L, Aydin Y, Erol K, Delibaşi T, Güler S (2010). Compatibility of different methods for the measurement of visceral fat in different body mass index strata. Diagn Interv Radiol.

[b6-jhk-47-73] Bolanowski M, Nilsson BE (2011). Assessment of human body composition using dual-energy x-ray absorptiometry and bioelectrical impedance analysis. Med Sci Monit.

[b7-jhk-47-73] Demura S, Sato S, Kitabayashi T (2005). Estimation accuracy of percent total body fat and percent segmental fat measured by single-frequency bioelectrical impedance analysis with 8 electrodes: the effect of difference in adiposity. J Sports Med Phys Fitness.

[b8-jhk-47-73] Erselcan T, Candan F, Saruhan S, Ayca T (2000). Comparison of body composition analysis methods in clinical routine. Ann Nutr Metab.

[b9-jhk-47-73] Esco MR, Snarr RL, Leatherwood MD, Chamberlain N, Redding M, Flatt AA, Moon JR, Williford HN (2014). Comparison of total and segmental body composition using DXA and multi-frequency bioimpedance in collegiate female athletes. J Strength Cond Res.

[b10-jhk-47-73] Gába A, Kapuš O, Cuberek R, Botek M (2014). Comparison of multi- and single-frequency bioelectrical impedance analysis with dual-energy X-ray absorptiometry for assessment of body composition in post-menopausal women: effects of body mass index and accelerometer-determined physical activity. J Hum Nutr Diet.

[b11-jhk-47-73] Glickman SG, Marn CS, Supiano MA, Dengen DR (2004). Validity and reliability of dual-energy X-ray absorptiometry for the assessment of abdominal adiposity. J Appl Physiol.

[b12-jhk-47-73] Jebb SA, Siervo M, Murgatroyd PR, Evans S, Frühbeck G, Prentice AM (2007). Validity of the leg-to-leg bioimpedance to estimate changes in body fat during weight loss and regain in overweight women: a comparison with multi-compartment models. Int J Obes (Lond).

[b13-jhk-47-73] Kitano T, Kitano N, Inomoto T, Futatsuka M (2001). Evaluation of body composition using dual-energy X-ray absorptiometry, skinfold thickness and bioelectrical impedance analysis in Japanese female college students. J Nutr Sci Vitaminol.

[b14-jhk-47-73] Lloret C, Ciangura C, Bouillot JL, Coupaye M, Declèves X, Poitou C, Basdevant A, Oppert JM (2011). Validity of leg-to-leg bioelectrical impedance analysis to estimate body fat in obesity. Obes Surg.

[b15-jhk-47-73] Lukaski HC, Siders WA (2003). Validity and accuracy of regional bioelectrical impedance devices to determine whole-body fatness. Nutrition.

[b16-jhk-47-73] Pietiläinen KH, Kaye S, Karmi A, Suojanen L, Rissanen A, Virtanen KA (2013). Agreement of bioelectrical impedance with dual-energy X-ray absorptiometry and MRI to estimate changes in body fat, skeletal muscle and visceral fat during a 12-month weight loss intervention. Br J Nutr.

[b17-jhk-47-73] Ravaglia G, Forti P, Maioli F, Boschi F, Cicognani A, Gasbarrini G (1999). Measurement of body fat in healthy elderly men: a comparison of methods. J Gerontol A Biol Sci Med Sci.

[b18-jhk-47-73] Spencer CE, Lingard JM, Bermingham MA (2003). Comparison of a footpad analyser with a tetrapolar model for the determination of percent body fat in young men. J Sci Med Sport.

[b19-jhk-47-73] Swartz AM, Swartz AM, Jeremy Evans M, King GA, Thompson DL (2002). Evaluation of a foot-to-foot bioelectrical impedance analyser in highly active, moderately active and less active young men. Br J Nutr.

[b20-jhk-47-73] Thivel D, O’Malley G, Peirera B, Duche P, Aucouturier J (2014). Clinical applicability of body and visceral adiposity indexes in severely obese youth. Am J Hum Biol.

[b21-jhk-47-73] Velazquez-Alva MC, Irigoyen-Camacho ME, Huerta-Huerta R, Delgadillo-Velazquez J (2014). A comparison of dual energy x-ray absorptiometry and two bioelectrical impedance analyzers to measure body fat percentage and fat-free mass index in a group of Mexican young women. Nutr Hosp.

[b22-jhk-47-73] Völgyi E, Tylavsky FA, Lyytikäinen A, Suominen H, Alén M, Cheng S (2008). Assessing body composition with DXA and bioimpedance: effects of obesity, physical activity, and age. Obesity (Silver Spring).

[b23-jhk-47-73] Wang JG, Zhang Y, Chen HE, Li Y, Cheng XG, Xu L, Guo Z, Zhao XS, Sato T, Cao QY, Chen KM, Li B (2013). Comparison of two bioelectrical impedance analysis devices with dual energy X-ray absorptiometry and magnetic resonance imaging in the estimation of body composition. J Strength Cond Res.

[b24-jhk-47-73] Wattanapenpaiboon N, Lukito W, Strauss BJ, Hsu-Hage BH, Wahlqvist ML, Stroud DB (1998). Agreement of skinfold measurement in Anglo-Celtic Australians. Int J Obes Relat Metab Disord.

